# Determining 3'-Termini and Sequences of Nascent Single-Stranded Viral DNA Molecules during HIV-1 Reverse Transcription in Infected Cells

**DOI:** 10.3791/58715

**Published:** 2019-01-30

**Authors:** Darja Pollpeter, Andrew Sobala, Michael H. Malim

**Affiliations:** 1 Department of Infectious Diseases, School of Immunology & Microbial Sciences, King's College London

**Keywords:** Genetics, Issue 143, 3'-terminus mapping, DNA polymerization, viral replication intermediates, HIV-1 reverse transcripts, unbiased ssDNA ligation, adaptor ligation, deep sequencing

## Abstract

Monitoring of nucleic acid intermediates during virus replication provides insights into the effects and mechanisms of action of antiviral compounds and host cell proteins on viral DNA synthesis. Here we address the lack of a cell-based, high-coverage, and high-resolution assay that is capable of defining retroviral reverse transcription intermediates within the physiological context of virus infection. The described method captures the 3'-termini of nascent complementary DNA (cDNA) molecules within HIV-1 infected cells at single nucleotide resolution. The protocol involves harvesting of whole cell DNA, targeted enrichment of viral DNA *via* hybrid capture, adaptor ligation, size fractionation by gel purification, PCR amplification, deep sequencing, and data analysis. A key step is the efficient and unbiased ligation of adaptor molecules to open 3'-DNA termini. Application of the described method determines the abundance of reverse transcripts of each particular length in a given sample. It also provides information about the (internal) sequence variation in reverse transcripts and thereby any potential mutations. In general, the assay is suitable for any questions relating to DNA 3'-extension, provided that the template sequence is known.

## Introduction

In order to dissect and understand viral replication fully, increasingly refined techniques that capture replication intermediates are required. In particular, the precise definition of viral nucleic acid species within the context of infected cells can provide new insights, since many viral replication mechanisms have to date been examined in isolated *in vitro* reactions. A prime example is the process of reverse transcription in retroviruses, such as human immunodeficiency virus 1 (HIV-1). The various steps of HIV-1 reverse transcription, during which the viral enzyme reverse transcriptase (RT) copies the single-stranded RNA genome into double-stranded DNA, have been studied primarily in primer extension assays with purified proteins and nucleic acids^1^,^2^,^3^,^4^,^5^. While fundamental principles were established, such assays do not incorporate all viral and cellular components and may not reflect biologically relevant stoichiometries of involved factors. Therefore, we designed a powerful technique to determine the spectra of reverse transcription intermediates with their precise cDNA 3'-termini (*i.e.,* determining their exact lengths) and nucleotide sequences in the context of infections of living cells^6^. Collection of data from time course experiments can be utilized to compare the profile of transcripts under various conditions, such as the presence of antiviral molecules or proteins, that may influence the efficiency and processivity of DNA synthesis and accumulation. This allows a more detailed understanding of the natural pathogen life cycle, which is often the basis for targeted drug design and successful therapeutic intervention.

**Figure 1:**
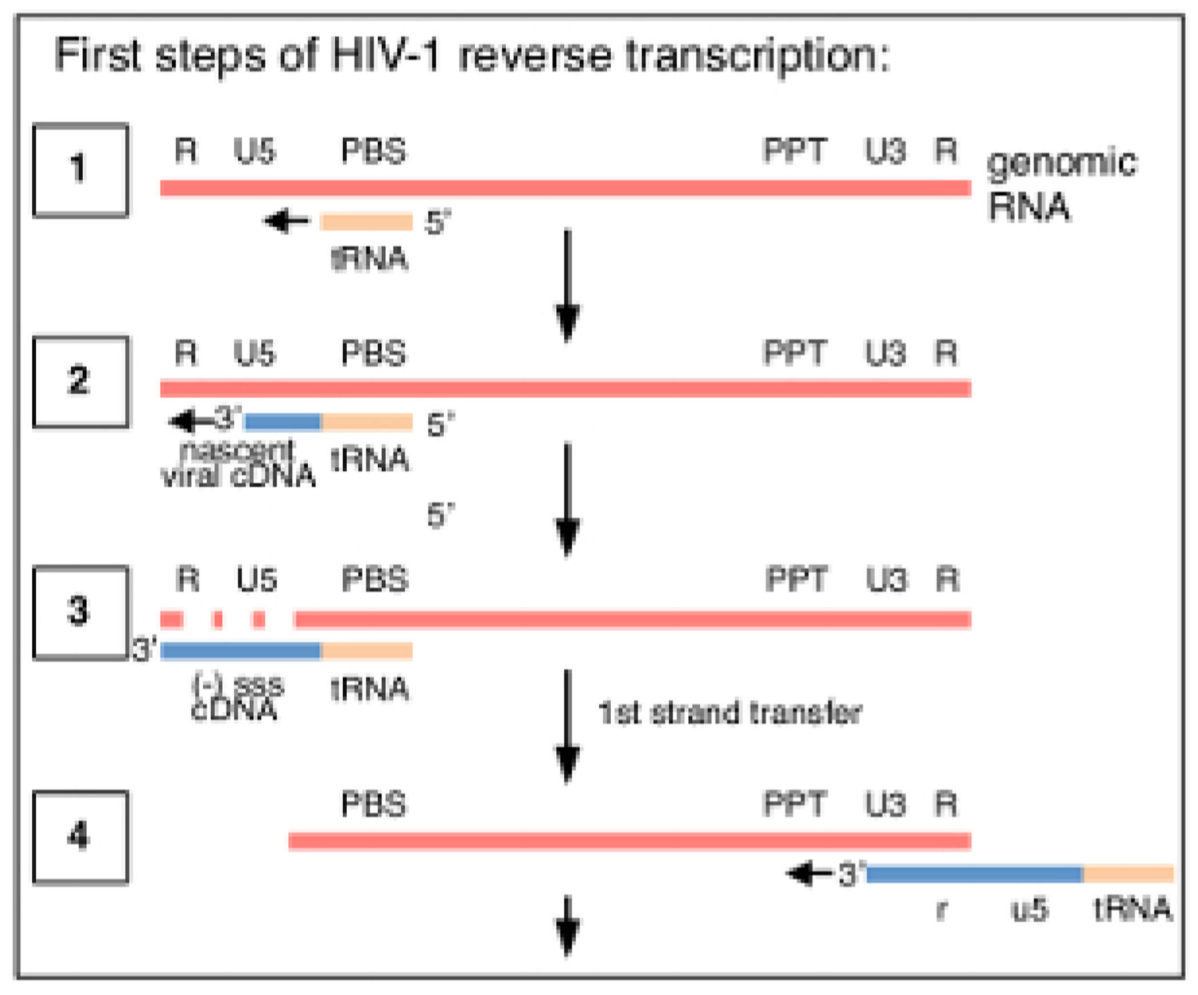
Diagram showing the first steps of HIV-1 reverse transcription. The process starts with annealing of tRNA(Lys,3) (orange) to the primer binding site (PBS) in the genomic viral RNA (step 1), which allows the initiation and elongation of viral cDNA (blue, step 2). Concomitantly, the template genomic RNA is degraded by RNaseH activity of RT (step 3). The first full intermediate in the process of reverse transcription is the minus-strand strong-stop (-)sss cDNA, which is complete when the RT catalyzed polymerization reaches the 5'-terminus of the gRNA repeat (R) region (step 3). The (-)sss intermediate is transferred to the 3'-terminus of the genomic RNA template by annealing to the complementary 3'-long terminal repeat (LTR) R region. From here, polymerization continues (step 4). In the described method the reverse transcription progression is determined by mapping the exact length of the nascent viral cDNA (blue). PPT, polypurine tract; U5, unique 5'-sequence; U3, unique 3'-sequence. This figure is republished from a previous publication^6^. Please click here to view a larger version of this figure.

**Figure 2:**
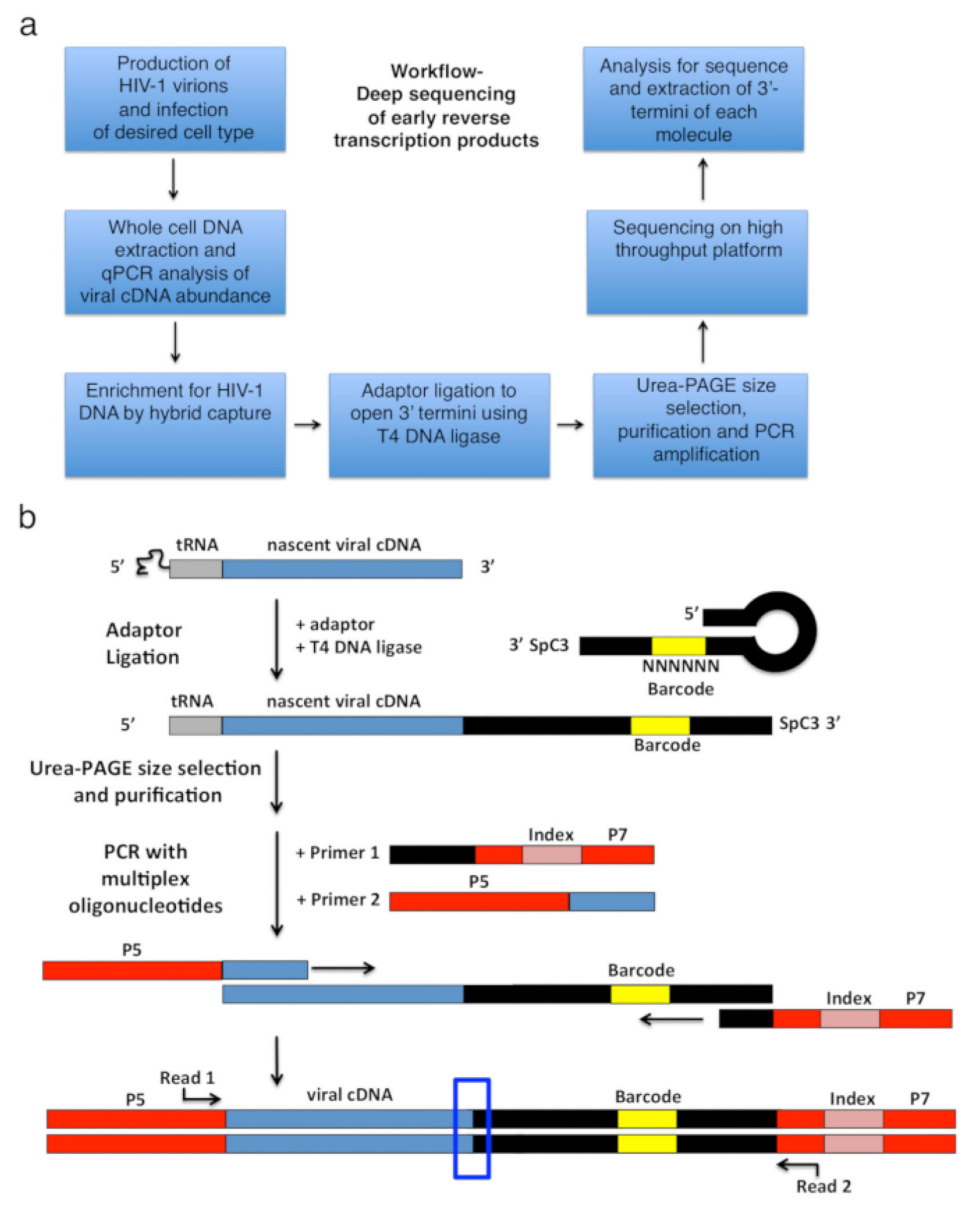
Workflow outline and schematics of the adapter ligation and PCR amplification strategy. (a) Workflow outlining main steps of the described technique to determine 3'-termini of HIV-1 reverse transcripts in infected cells. The figure is adapted from a previous publication^6^. (b) Schematic of the adaptor ligation and PCR amplification strategy. Nascent cDNA molecules of varying length that have been purified in previous steps are ligated to a single-stranded DNA adaptor using T4 DNA ligase. The hairpin adaptor (named "full Kwok + MiSeq", see Table 1 design was inspired by Kwok *et al*.^11^. The adaptor carries a random 6 nt barcode sequence, which allows for base-pairing to facilitate ligation and simultaneously serves as an identifier for unique reads. The 3'-termini of the adaptor carries a spacer (SpC3) to prevent self-ligation. Ligated products are separated from excess adaptor by denaturing polyacrylamide gel electrophoresis (PAGE). Nucleic acids in the gel are stained and cut into three separate, equal-size gel pieces in the area from above the adaptor to the well as done in^25^. After elution, precipitation, and resuspension, the products are PCR amplified with primers annealing to the known sequence of the adaptor (primer 1, multiplex oligonucleotide kit, see Table of Materials) and a primer carrying the first 22 nt of the HIV-1 5'-LTR sequence immediately following the tRNA (primer 2, MP1.0 + 22HIV). The 5'-termini of the chosen primers carry adaptors for the chosen sequencing platform (P5 and P7) as well as an index sequence to distinguish individual samples run in the same library. Starting points of the sequencing read primers are indicated. The blue box indicates the region of interest to determine the original 3'-termini of the captured molecule. This figure is adapted from a previous publication^6^. Please click here to view a larger version of this figure.

HIV-1 reverse transcription comprises a series of successive events initiated by annealing of a tRNA primer to the genomic RNA template, which is then extended by RT to produce a short single-stranded cDNA transcript called a minus-strand strong-stop (-sss) (see Figure 1). Subsequently, the -sss cDNA is transferred from the 5'-long terminal repeat (LTR) to the 3'-LTR of the genomic RNA, where it anneals and serves as the primer for continued RT mediated elongation of the minus strand DNA (see reviews on reverse transcription^1^,^2^,^3^,^4^). This first strand transfer is one of the rate-limiting steps of reverse transcription; hence, the -sss cDNA is known to accumulate. The basic workflow and library design to capture the reverse transcription products in infected cells is outlined in Figure 2a. The specific primers and analysis settings that are used in the protocol and listed in Table 1 target all early reverse transcription cDNA intermediates within the length range of 23 to ~650 nt, which includes the 180-182 nt -sss DNA. However, appropriate minor adaptations to the strategy will allow application to not only late reverse transcription products but also other viruses and systems, where the objective is to detect 3'-OH containing DNA ends. Important limitations to consider include the length range of the final PCR product in the library; in particular, templates in which the distance between the adaptor on the open 3'-terminus and the upstream primer site exceed ~1000 nt will likely be less efficiently sequenced, potentially introducing misleading technical biases during library preparation (see discussion for more details and adaptation suggestions).

Previously reported techniques for the systematic determination of 3'-termini of nucleic acid strands have focused on RNA, not DNA, molecules. One example is 3' RACE (rapid amplification of cDNA ends)^7^, which relies on the polyadenylation of mRNA. In addition, adaptor ligation-based strategies employing RNA ligases were developed, which have included RLM-RACE (RNA ligase-mediated RACE)^8^ or LACE (ligation-based amplification of cDNA ends)^9^. It is important to emphasize that ligation-based amplifications are sensitive to any bias introduced by the ligation reaction itself. For example, ligation may be more or less efficient depending on a particular nucleotide in the 3' position, the sequence, total molecule length, or local structure. Such ligase preferences lead to incomplete capture of molecules and misrepresentation in the readout, which we and others have observed^9^,^10^. To minimize ligation bias during the adaptor addition steps in the protocol described herein, we tested a number of ligation strategies and found the use of T4 DNA ligase with a hairpin single-stranded DNA adaptor (as described by Kwok *et al*.^11^) to be the only procedure with near quantitative ligation that did not result in significant differences in ligation efficiency when assessed with a specifically selected set of control oligonucleotides^6^. The choice of this ligation strategy is, therefore, a key feature in the success of this protocol.

To date, monitoring of HIV-1 RT progression in infected cells has primarily been accomplished by measuring reverse transcription products of various length with quantitative PCR (qPCR) using primer-probe sets that uniquely measure shorter or longer (early and late, respectively) cDNA products^12^,^13^,^14^. While this qPCR approach is appropriate to determine intrinsic efficiencies of the reverse transcription process in cellular systems, the output is of relatively low resolution, with no sequence information being derived. Our new approach, based on optimized adaptor ligation, PCR-mediated library generation, and deep sequencing addresses the technology gap and offers an opportunity to monitor reverse transcription during HIV-1 infection quantitatively and at single nucleotide resolution.

We have illustrated the utility of this method in a study that distinguished between two proposed models for the capacity of the HIV-1 restriction factor APOBEC3G (apolipoprotein B mRNA editing enzyme catalytic polypeptide-like 3G) to interfere with the production of viral reverse transcripts^6^.

## Protocol

NOTE: Please refer to the Table of Materials for specific reagents and equipment used in this protocol.

### 1 Virus Production and Cell Infection

CAUTION: Infectious HIV-1 should only be handled in approved biosafety containment laboratories.

NOTE: Production of HIV-1 particles by transient transfection of human embryonic kidney (HEK) 293T cells, as outlined in step 1.1, is a standard procedure and has been described previously^15^,^16^. General cell culture procedures are described previously^17^.

1. HIV-1 virus production.
  1. Maintain 293T cells in Dulbecco's modified Eagle medium (DMEM) supplemented with 10% fetal bovine serum (FBS) and 1% penicillin/ streptomycin (full DMEM) in a standard cell culture incubator at 37 °C and 5% CO_2_ as described previously^17^.
  2. In a standard laminar flow tissue culture hood remove the growth media and add 3 mL of pre-warmed (37 °C) trypsin to a near-confluent 10 cm cell culture dish (~1.2 x 10^7^ cells) of 293T cells. Put the dish back in the incubator for 2-3 min.
  3. Take the dish from the incubator back into the tissue culture hood and add 7 mL of full medium. Pipette up and down within the dish several times to resuspend the cells. Split the cells 1:4 by adding 2.5 mL of the cell suspension to a new 10 cm dish and fill it with 7.5 mL of full medium.
  4. The next day, mix 10 µg of proviral HIV-1 plasmid DNA (such as pNL4.3) with 1 mL of serum-free minimal essential medium and add polyethylenimine (PEI) solution (25,000 mW, 1 mg/mL pH 7) at 4.5 µL per 1 µg DNA. Incubate for 10 min at RT and add dropwise to the 293T cells.
  5. 24 h after transfection, remove the medium and replace it with 6 mL of full DMEM containing RNase-free DNase at 20 U/mL medium. After 6 h, replace the medium with 10 mL of full DMEM.
  6. 48 h after transfection, harvest the supernatant and filter it through a 0.22 µm filter, using a 10 mL syringe, into a 15 mL polypropylene tube.
  7. Add 2 mL of sterile 20% sucrose in 1x phosphate buffered saline (PBS) to an open-top thin-walled ultracentrifuge tube. Slowly overlay the sucrose with the filtered cell supernatant.
  8. Centrifuge for 1 h 15 min at 134,000 x g at 4 °C using an ultracentrifuge.
  9. Remove the tubes from the ultracentrifuge carefully. Slowly take off both the supernatant and sucrose using a suction or a pipette. Use a smaller pipette and tilt the tube when taking the last sucrose solution out. Leave the pelleted virus in the bottom of the tube.
    NOTE: The pellet will not be visible.
  10. Add 200 µL of 1x PBS, leave in the fridge for 4 to 12 h, resuspend, and freeze in 20 µL aliquots at -80 °C.
  11. Determine p24^Gag^ content using the a p24 HIV-1 antigen ELISA kit (following manufacturer's instructions).
2. T-cell line infection.
  1. Culture an immortalized T-cell line (*e.g*., CEM-SS cells) in Roswell Park Memorial Institute (RPMI) 1640 medium supplemented with 10% FBS and 1% penicillin/streptomycin (full RPMI). Count the cells using a hemocytometer^18^ and seed 1 well per sample with 1 mL of full RPMI with 2 x 10^6^ cells/mL in a 12-well format cell culture plate.
  2. Add HIV-1 particles equivalent to 150 ng p24^Gag^ and place the plate into a swinging centrifuge bucket with biocontainment lids to spin-infect by centrifugation in a benchtop centrifuge for 2 h at 2,000 x g at 30 °C.
  3. Remove plate from the centrifuge and let it rest for 1 h in a standard tissue culture incubator at 37 °C and 5% CO_2_.
  4. To wash off input virus, collect cells by transferring the cell suspensions to microcentrifuge tubes and centrifuging in a microcentrifuge at RT (RT) at 500 x g for 2 min. Take off the supernatant without disturbing the cell pellet.
  5. Resuspend the cell pellets in 1 mL of pre-warmed (37 °C), sterile 1x PBS. Repeat the centrifugation, supernatant removal, and resuspension steps two more times.
  6. Centrifuge again, remove the supernatant and resuspend the cell pellets in 1 mL of full RPMI. Add each suspension to one well in a new 12 well plate.
  7. At 6 h post-initial addition of virus (4 h post-centrifugation), harvest the cells by centrifugation as done in step 1.2.4. Remove and discard the supernatant. The cell pellets can be frozen at -80 °C or processed directly for DNA extraction.

### 2 DNA extraction, HIV-1 DNA Quantification, and Enrichment by Hybrid Capture

1. Extract whole cell DNA with a blood and tissue total DNA extraction kit by following the kit manual for tissue culture cells. The only change is elution in 200 µL of nuclease-free H_2_O instead of the provided elution buffer.
  NOTE: After addition of the chaotropic lysis buffer (the kit's "AL buffer") and proteinase, samples can be removed from the biosafety containment laboratory and handled in a standard safety level laboratory for the rest of the protocol.
2. Determine the copy number of HIV-1 cDNA by qPCR.
  1. Take 17 µL of the eluate from step 2.1 and add 2 µL of 10x restriction enzyme buffer together with 1 µL of DpnI restriction enzyme. Incubate for 1 h at 37 °C to remove any potential residual input plasmid DNA from transfection.
  2. Carry out qPCR for minus-strand strong-stop cDNA using the following primer probe set: oHC64 (5′-taactagggaacccactgc-3′) and oHC65 (5′-gctagagattttccacactg-3′) and probe oHC66 (5′-FAM- acacaacagacgggcacacacta-TAMRA-3′). qPCR setup and exact conditions can be found in the references^6^,^13^. Carry along samples with a serial dilution of pNL4.3 proviral plasmid as a standard curve to determine copy numbers of cDNA molecules.
    NOTE: See discussion for expected quantities.
3. HIV-1 DNA enrichment by hybrid capture.
  NOTE: From this step forward, it is preferable to use microcentrifuge tubes with low nucleic acid binding properties as well as aerosol filter pipette tips for all DNA samples. If possible, work in a PCR workstation. All steps and reagents are at RT (RT) unless otherwise stated.
  1. To prepare a master mix of magnetic streptavidin beads, pipette 100 µL beads per sample into a single microcentrifuge tube. Place the tube on a magnet suitable for microcentrifuge tubes.
  2. After the beads have settled towards the magnet side of the tube (~1 min), take off the storage buffer, remove the tube from the magnet and resuspend beads in 500 µL of bind and wash buffer (BW buffer, 5 mM Tris-HCL pH 7.5, 0.5 mM EDTA, 1 M NaCl) to wash.
  3. Place the tube back on magnet, remove the supernatant and add 500 µL casein solution. Take of the magnet, resuspend and incubate for 10 min at RT, then wash with BW buffer.
    NOTE: A wash refers to placing the tube on the magnet, taking off the supernatant, taking the tube off the magnet, adding the buffer, and resuspending.
  4. Place tube back on the magnet, take off the supernatant and resuspend beads in 500 µL of BW buffer. Add 50 pmol of each capturing biotinylated oligonucleotides (see Table 1, three oligos in this case) per sample. (For example, if 5 DNA samples are to be processed, use 500 µL of magnetic beads from step 2.3.1 and 250 pmol of each oligonucleotide).
  5. Incubate for 30 min at RT while rocking in an end-over-end mixer.
  6. Wash beads with the immobilized oligonucleotides two times with 500 µL of 1x TEN buffer (10 mM Tris HCl pH 8.0, 1 mM EDTA, 100 mM NaCl).
  7. Resuspend beads in 10 µL of 1x TEN buffer per sample.
  8. For each sample label one microcentrifuge tube and add 10 µL of beads suspension, 170 µL of DNA (from step 2.1) and 90 µL of 3x TEN buffer. Incubate in a dry heat block at 92 °C for 2 min to denature the DNA.
  9. Move the tubes to a different dry heat block, which is set to 52 °C, and incubate for 1 h. Invert to mix regularly (~every 10 min) during this incubation.
  10. Wash once with 500 µL of 1x TEN buffer and resuspend in 35 µL nuclease-free H_2_O.
  11. To elute, incubate the tubes at 92 °C in a dry heat block for 2 min. Then, quickly move the tubes onto the magnet (one tube at a time). Once beads are bound to the side of the tube, transfer the supernatant containing the HIV-1 DNA to a fresh tube.
  12. Optional: Repeat qPCR (as done in step 2.2.2) to determine the recovered HIV-1 cDNA.
    NOTE: See discussion for expected quantities.

### 3 Adaptor Ligation

1. Preparing the adaptor
  1. Resuspend lyophilized adaptor (see Table 1 "full Kwok + MiSeq") at 100 µM in nuclease-free H_2_O.
  2. Per sample plus one control sample, combine 0.45 µL of 10x T4 DNA ligase buffer, 4 µL of adaptor, and 0.05 µL of nuclease-free H_2_O. Heat to 92 °C for 2 min and let each cool down slowly.
    NOTE: If the option is available use a PCR machine with adjustable cooling rate (use 2% rate). This takes about 30 min from 92 °C to 16 °C. Alternatively, use a dry heat block at 92 °C and turn off. Take the adaptor mastermix out when the heat block is back at RT. This is to let the adaptor form a hairpin structure (see Figure 2b).
2. Prepare a control reaction with a set of synthesized oligonucleotides (see Table 2) instead of DNA extracted from cells.
  1. Make stocks of 100 µM of each oligonucleotide. Mix 1 µL of each of the 17 oligonucelotides and add 8 µL of H_2_O for an equimolar ratio in a final volume of 25 µL.
  2. Dilute the mix 1:2,500 in nuclease-free H_2_O in a serial dilution. Combine 1 µL of the mix with 17.3 µL of nuclease-free H_2_O to use in the control sample ligation in step 3.3.1 so that each oligonucleotide is present at 1.6 fmol (equivalent to 0.026 nM in the 60 µL reaction).
3. Setting up ligations
  1. For 60 µL final volume reactions in PCR tubes combine 6 µL of 10x T4 DNA ligase buffer, 24 µL of 40% PEG, 6 µL of 5 M betaine, 4.5 µL (400 pmol) of adaptor (pre-annelead as in step 3.1.2), 1.2 µL of T4 DNA ligase (2,000,000 Units/mL) and 18.3 µL of DNA (from step 2.3.11)
    NOTE: Take special care with viscous solutions such as 40 % PEG to maintain accurate volumes. Do not make a mastermix.
  2. Set up the same reaction as performed in step 3.3.1 but with the control oligonucleotide mix prepared in step 3.2.2.
  3. Mix the reactions well and incubate in a PCR machine at 16 °C overnight.

### 4 Adaptor Removal and Size Separation

1. Denaturing gel electrophoresis
  1. Add 30 µL of formamide-containing DNA gel loading buffer to each ligation reaction. Mix well by pipetting.
  2. Heat for 2 min at 94 °C in PCR machine, then immediately put on ice.
  3. Place a precast 6% Tris/borate/EDTA (TBE) denaturing urea polyacrylamide gel (10-well comb) in an appropriate gel tank. Add 1x TBE (89 mM Tris-base, 89 mM boric acid, 2 mM EDTA) running buffer and pre-run the gel for 20 min at 250 V/max constant.
  4. Wash out the gel pockets with running buffer using a syringe and 21G needle.
  5. Load each 90 µL sample into three wells (30 µL per well) and run for 20 min (250 V/max) until the dark-blue dye front is about halfway through the gel.
2. Staining and cutting nucleic acids from the gel
  1. Prepare 3 small microcentrifuge tubes (0.5 mL) per sample by poking holes into the bottom using a 21 G syringe needle (take caution while working with sharps). Insert each of the prepared tubes into a 2.0 mL microcentrifuge tube and label them with the sample name plus "low", "mid" or "high".
  2. Take out and pry open the gel cassette. Cut the gel vertically with a razor blade to generously excise the strip with the 3 wells of loaded samples. Add the gel strip to a container with 1x TBE (about 30 mL) and 5 µL of cyanine nucleic acid stain. Incubate for 3-5 min.
    NOTE: The gel extraction step is particularly sensitive to cross-contamination. It is advisable to only run 1 sample per gel and using a separate, clean container for each gel staining. Gloves should be changed if gel particles contact gloved fingers.
  3. Clean the surface of a blue light transilluminator thoroughly with ddH_2_O. Take the gel piece out of the staining container and add it to the light box.
  4. Turn on the light box and inspect the stained nucleic acids through the orange filter.
    NOTE: The adaptor typically appears overloaded and runs as big "blob" with ligated HIV-1 DNA running above as a streak.
  5. Using a new razor blade, cut away the sides of the gel if there are areas with no sample loaded still present. Next, cut just above the adaptor to remove the adaptor and lower gel parts. Finally, cut away the very top of the gel including about 1 mm of the gel pockets, which often have a sharp intense signal of higher molecular weight DNA.
  6. Divide the remaining gel piece containing the sample, which is typically ~2 x 3 cm in size, horizontally into three even pieces: "low", "mid" and "high" molecular weight areas.
    NOTE: Each piece will now be handled separately [*i.e*., there will be three tubes (low, mid, and high)] per original sample.
  7. Cut each of the three gel fragments into smaller pieces (~2 x 2 mm particles) and transfer them into the prepped 0.5 mL microcentrifuge tubes (step 4.2.1).
  8. Spin at top speed with open lids for 1 min to squeeze the gel pieces through the hole into the 2 mL tube to create a gel slush. If any gel particles remain in the bottom of the 0.5 mL tube, transfer them to the 2 mL tube manually using a needle or pipette tip.
3. DNA extraction
  1. Add 1 mL of urea gel extraction buffer (0.5 M NH_4_CH_3_CO_2_, 1 mM EDTA, 0.2% SDS) to the gel slush. Rotate tubes for a minimum of 3 h (overnight is acceptable) at RT with an end-over-end mixer.
  2. Use a clean set of tweezers to add one small round glass fiber filter to centrifuge columns with cellulose acetate membrane filters (0.2 µm), which avoids membrane clogging. Put the filter in place with an inverted pipette tip.
  3. Briefly spin the 2 mL tubes with gel slush and extraction buffer in a microcentrifuge and transfer 700 µL of the supernatant to the prepared filter columns. Keep the gel slush and remaining supernatant.
  4. Centrifuge the filter columns in a microcentrifuge at top speed for 1 min. Transfer the flowthrough into a new 2.0 mL microcentrifuge tube.
  5. Reload the columns with the remaining supernatant. Try to obtain as much liquid as possible from the extraction slush. Transfer of gel pieces is not a concern. Spin again and combine flowthroughs of the same extraction samples.
4. DNA precipitation
  1. Add 3 µL of polyA RNA (1 µg/µL; as a carrier), 1 µL of glycogen, and 0.7 mL of isopropanol to the flowthrough from step 4.3.5. Vortex briefly and freeze at -80 °C overnight.
  2. Take samples out of the -80 °C freezer and let them thaw briefly. Put them in a cooled (4 °C) microcentrifuge and spin for 30 min at top speed.
  3. Remove and discard the supernatant. Be very careful not to remove the pellet. Leave 30 to 50 µL of liquid if it is uncertain that pellet would be removed otherwise.
    NOTE: Typically all "high" samples show a more visible pellet than "mid" and "low" samples.
  4. Add 800 µL of 80% ethanol. Invert tubes and spin again for 1 min at top speed. Remove the majority of ethanol with a pipette, briefly spin the tubes again, and remove more ethanol with a smaller volume pipette.
  5. Let any remaining ethanol evaporate by placing the tubes with an open lid into a 55 °C dry heat block. When samples are dry (2-4 min) add 20 µL of nuclease-free H_2_O and spread around the bottom of the tube to ensure the DNA pellet is dissolved. The DNA sample can be stored at -20 °C.

### 5 PCR Amplification and Library Preparation

1. Set up a 40 µL PCR reaction with 20 µL of DNA polymerase pre-mix, 18 µL of precipitated and redissolved DNA from step 4.4.5, 1 µL of forward primer "MP1.0 + 22HIV" (10µM) (see Table 1), and 1 µL of multiplex oligo primers (index primers 1 to 24) (see Table of Materials).
  NOTE: Run the three reactions (low, mid, high) of each sample in separate PCR reactions, but with the same indexed primer. Use a different index for each of the original infection samples.
  1. Run the PCR reactions under the following conditions: 2 min at 94 °C denaturation, then 18 cycles of 3-step PCR; 15 s at 94 °C denaturation, 15 s annealing at 55 °C, and 30 s extension at 68 °C.
2. As a quality control option, analyze PCR reactions with high sensitivity automated gel electrophoresis system. Take 2 µL of a low, mid, and high sample to run as per manufacturer's instruction.
  NOTE: The two primers should be visible and often run at a calculated length of about 45 and 95 nt (actual length differs). Additionally, DNA should be detected between 150 to 500 nt. If no signal is present, it is advisable to add additional PCR cycles, between 2 and 10 additional cycles. Do not add additional cycles for the oligonucleotide control samples created in step 3.3.2.
3. To remove the primers use a paramagnetic bead-based PCR clean-up system.
  1. Take 20 µL of each PCR reaction and pool the samples together (mix all the samples at this point). Freeze the remaining 20 µL reactions as backups at -20 °C.
  2. Let the paramagnetic beads come to RT and mix the pooled PCR reactions with 1.8x the volume of the bead solution. Mix by pipetting and incubate for 5 min.
    NOTE: As an example, if 4 samples were prepared and each have low, mid, and high reactions, the volume would be 4 x 3 x 20 µL = 240 µL PCR reactions with a 432 µL bead solution.
  3. Put the tubes on a microcentrifuge tube magnet, let the beads bind for ~1 min, and take off the supernatant to discard. Leave the tubes on the magnet and add 500 µL of 80% ethanol.
  4. Leave the ethanol for 30 s, then take off thoroughly and let the beads airdry for ~5 min. Add 40 µL of nuclease-free H_2_O, take the tubes off the magnet, and pipette up and down multiple times.
  5. Leave the suspension for 5 min. Put the tube back on the magnet, let the beads settle to the side, and transfer the supernatant to a new tube. This is the library. Take a 10 µL aliquot for quality controls and freeze the rest at -20 °C.

### 6 Evaluating the Library

1. Determine the library quality, concentration, and molarity.
  1. Use a fluorometric quantification method. Measure 1 µL and 3 µL of the library with a high sensitivity dsDNA assay kit as per manufacturer's instructions.
    NOTE: Typical concentrations are between 1 and 10 ng/µL.
  2. Measure the library DNA molecular weight spectrum by high sensitivity automated gel electrophoresis as described above (step 5.2).
  3. Use the automated gel electrophoresis analysis to determine the average molecular weight of the library and calculate to dilute the library in nuclease-free H_2_O to 4 nM. Several libraries can be combined as long as all indices are unique.
2. Optional low throughput quality control
  1. Subject the DNA library to TA cloning^19^ to insert library molecules into vectors for amplification. Follow the kit's instructions, grow out ~10-20 colonies and extract DNA *via* miniprep protocols, as described here^20^.
  2. Sequence vector using local sequencing services and check that the inserts contain the desired HIV-1 derived sequences and library-specific adaptors.

### 7 High-Throughput Sequencing Run

1. Create a sequencing sample sheet with the commercial software provided with the sequencing platform.
  1. Indicate the selected sequencing kit. Typically, choose a 150-cycle kit, but others are suitable depending on the desired read length.
  2. Select "Fastq only" as the application workflow. Choose one of the templates that contains the 24 indices present in the multiplex oligonucleotide kits (indicated in kit manual).
  3. Select "25 nt" for Read1 and "125 nt" for Read2. Keep 6 nt for single index read.
    NOTE: In the in-house analysis only Read2 is used in the analysis. Keep Read1 at a minimum of 25 nt for sequencing platform algorithm purposes.
2. Follow manufacturer's instructions precisely for the pre-run library preparation and setup. Opt for the maximum 20 pM concentration and use a 15% PhiX spike, as the library is of very low complexity.

### 8 Data Analysis

1. Check if the pass filter percentage and average Q30 quality score are acceptable as per sequencing platform manufacturer's guidelines.
  NOTE: Pass filter is typically > 90% and Q30 scores are typically > 80%.
2. Download the .fastq.gz files from the manufacturer's sequencing hub.
3. Setting up the sequencing script
  1. Create a new directory (folder) named "AnalysisXYZ" and go to https://github.com/malimlab/seqparse to download all source code files (parse_sam.pl, rc_extract.pl, parse.sh) into this directory.
  2. Download the short-read aligner Bowtie, version 1.1.2, from http://bowtie-bio.sourceforge.net/index.shtml into the same directory.
  3. The download creates a subdirectory within "AnalysisXYZ" named "Bowtie-1.1.2". Within this directory open subdirectory "Indexes" and download the provided template sequences consisting of 6 files with .ebwt extensions.
  4. Download the FASTQ/A short reads pre-processing toolkit fastx-0.0.13 from http://hannonlab.cshl.edu/fastx_toolkit/download.html into the "AnalysisXYZ" directory.
  5. Download both Samtools (https://sourceforge.net/projects/samtools/files/) and bam-readcount (https://github.com/genome/bam-readcount) into the "Documents" directory.
4. Move the .fastq.gz files, downloaded in step 8.2, of all read 2s (ending in …_R2_001.fastq.gz) into the "AnalysisXYZ" directory.
5. Open the command console/terminal. Move to the "AnalysisXYZ" as the current directory using cd commands. Type "./parse.sh ." to run the scripts.
6. Find the .csv files with summaries for all samples on total read counts, length adjusted read counts, and normalizes read counts, as well as files with the base variation, for each sample in a directory named parse_results within the "Analysis XYZ" directory.
  NOTE: See the discussion for further information about the analysis process. The script returns csv files with total reads for each nucleotide along the HIV-1_NL4.3_ strong-stop sequences and first strand transfer until nucleotide 635. As a guidance, 50,000 to 100,000 unique reads are typically observed in samples from infections with the indicated cell numbers and viral inocula and without antiviral proteins or compounds. The oligonucleotide control sample usually produces 100,000 to 200,000 reads.

## Representative Results

**Figure 3:**
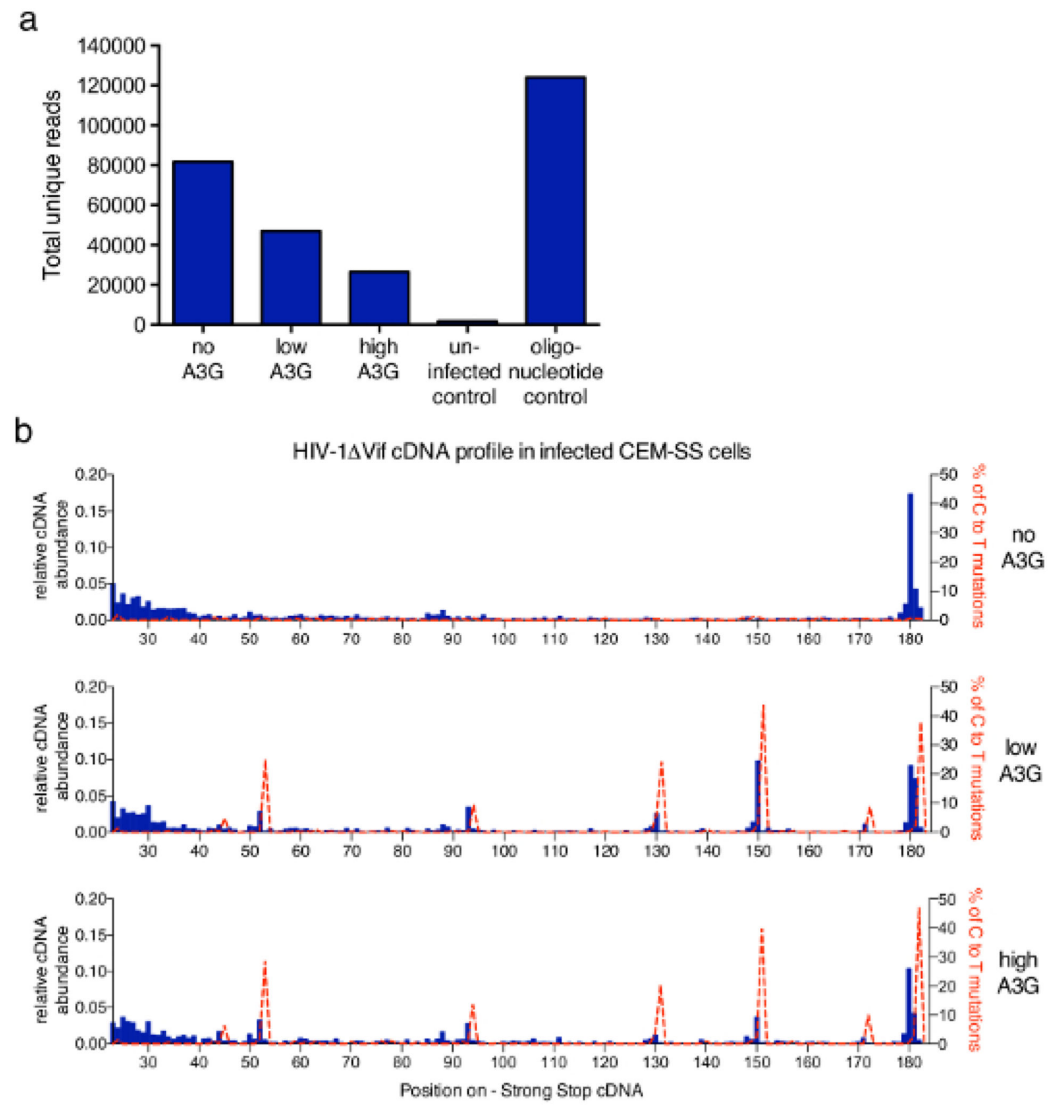
Representative results. (a) The total read count of representative samples processed with the described protocol. This includes all sequences that were identified as unique reads of HIV-1 molecules with their 3'-termini within the first 635 nt of the minus strand cDNA (up to the PPT, see Figure 1). Infection with HIV-1 not carrying A3G yields the highest number of reads, whereas A3G inhibits cDNA synthesis and thereby reduces the total read count. Uninfected cells served as a negative control, while a set of synthetic oligonucleotides provides a positive control. b) The relative abundance of cDNAs for each length between nt positions 23 and 182 (full-length -sss cDNA is 180 to 182 nt) of the HIV-1_NL4.3_ sequence (x-axis) is shown in blue histograms (scale on the left y-axis). The relative abundance of cDNA was calculated from the absolute number of sequences terminating at a given nucleotide within the -sss cDNA sequence divided by the sum of all reads measuring 182nt or less. Shown in dashed red lines are the percentages of reads carrying C-to-T/U mutations at the respective position (scale on the right y-axes). Figure 3b is republished from a previous publication^6^. Please click here to view a larger version of this figure.

The technique described in this article was applied to a wider study to address the mechanisms underlying inhibition of HIV-1 reverse transcription by the antiretroviral human protein APOBEC3G (A3G)^6^. Figure 3 shows representative results obtained after employing the protocol in samples from CEM-SS T-cells infected with *vif*-deficient HIV-1 in the absence or presence of A3G. The total number of unique reads obtained from each sample after filtering out any PCR duplicates that have the same 6 nt barcode and the same length (performed by the analysis software provided) are plotted in Figure 3a. Increasing levels of A3G reduce the total read number reflecting the inhibitory effect of A3G on RT mediated cDNA synthesis previously described and measured by qPCR^6^,^13^,^21^,^22^. In Figure 3b, the fraction of molecules at each possible length within the first 182 nt are shown. For HIV-1 infection in the absence of A3G, the most abundant species is the main 180 nt strong-stop molecule itself, with some accumulation of reads in the shorter range (23 to 40 nt) (top graph, blue histograms). The addition of A3G changes this profile as a sharp increase of shorter, truncated cDNA molecules at a few very specific, reproducible positions is detected (middle and lower graphs). Since A3G is a cytidine deaminase, cytosine-to-uridine (identified as C-to-T) mutations in the cDNA occur when A3G is present in the infecting virions^21^,^23^,^24^. Using the obtained sequencing information, the percentage of C-to-T mutations was plotted on the same graph (red dotted line). It should be noted that the mutational profile is derived from all unique reads combined and coverage of each nucleotide will vary. However, if required sequence information can be related back to each molecule and correlated with a specific 3'-terminus. The data provided were taken from Pollpeter *et al.*^6^ and the correlation between mutational and cDNA length profiles was demonstrated to be due to detection and cleavage of deaminated cDNA by the cellular DNA repair machinery.

**Figure 4:**
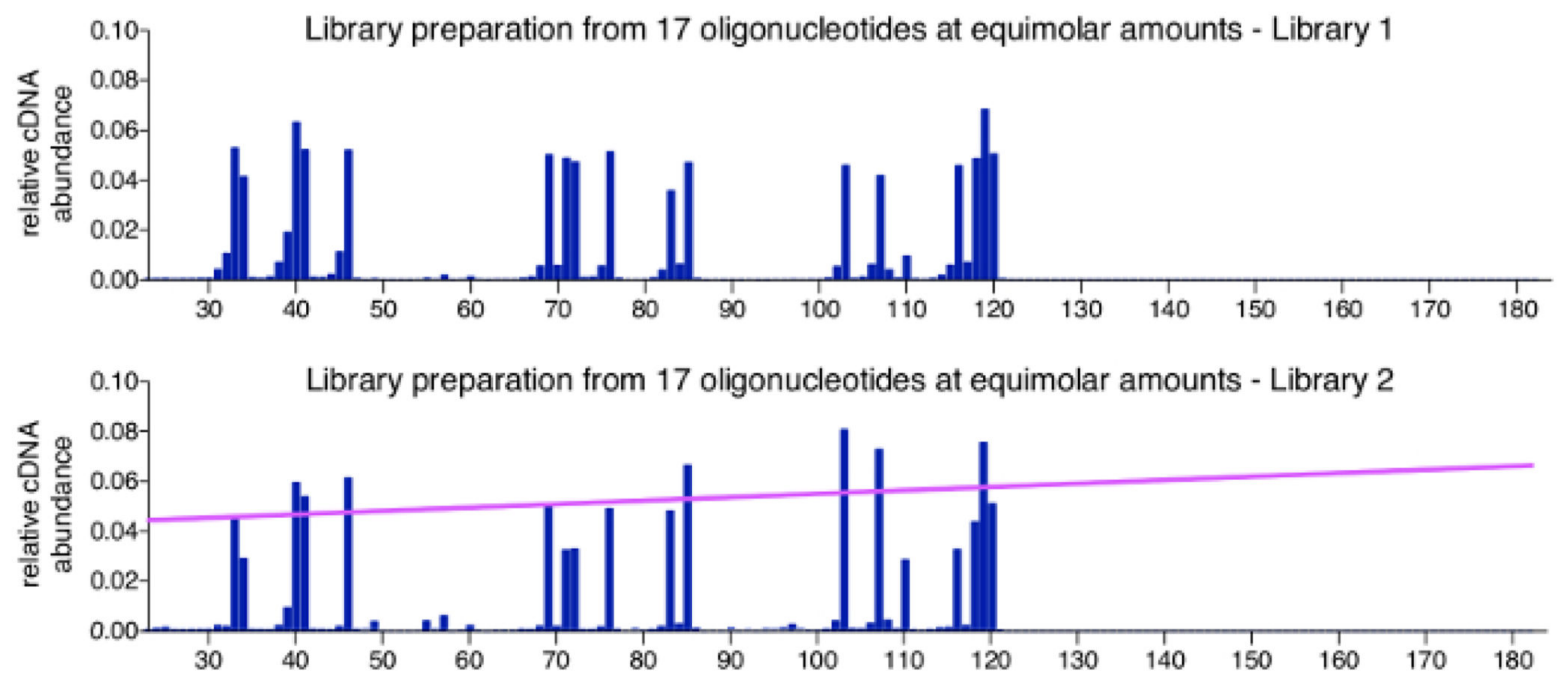
Representative results of control samples. Shown are two profiles for pools containing equimolar amounts of 17 different length synthetic oligonucleotides. These oligonucleotides have sequences from HIV-1_NL4.3_ and were selected to cover various lengths and present all 4 bases as a 3'-nucleotide (see Table 2). The top graph shows the positive control sample from Figure 3a. No significant bias towards molecule length or the open 3'-termini is detected. The bottom graph shows a different library run, which produced a minor length bias in sequencing. In this case, it is advisable to apply a normalization factor, which is derived from the slope (shown in pink) that represents the size bias. This figure is republished from a previous publication^6^. Please click here to view a larger version of this figure.

A positive control for the 3'-mapping approach can easily be produced by processing a pool of synthetic oligonucleotides of known sequence, length, and concentration. This control is added at the adaptor ligation in step 3.3.2 and advised to be included in all multiplexed libraries. Data obtained from a control sample should have all the oligonucleotides at the expected input ratios, with only very minor background reads. Figure 4 shows results of a positive control set of 17 chemically synthesized oligonucleotides (for sequences, see Table 2), which were mixed at equimolar ratios. As expected, all molecules appear in close to equal abundance with only small variations (top graph). While most positions within the -sss DNA sequence that were not represented by an oligonucleotide return zero read counts, we observed minor species that are 1 or 2 nt shorter than the actual control oligonucleotides. We have not further investigated these minor species but assume that they represent degraded or incomplete products potentially present in the provided oligonucleotide stocks at purchase (oligonucleotides were ordered as HPLC purified, for which the manufacturer indicates > 80% purity). The bottom graph shows the control sample from a different library run, where variation is slightly higher between the 17 oligonucleotides and correlates with overall length in that longer control molecules are detected more efficiently then shorter ones. This may be due to a minor bias in PCR reactions or in clustering during MiSeq sequencing, which has an insert size optimum and may occur with libraries carrying particularly broad insert ranges. A basic way to address this bias is the application of a normalization factor based on the slope that indicates the bias correlating to molecule length (pink line). The required calculations are included in the analysis program (see step 8.3 in the protocol).

## Discussion

The availability of fast, reliable, and cost-effective deep sequencing has revolutionized many aspects in the field of life sciences, allowing great depth in sequencing-based analyses. A remaining challenge lies in the innovative design and creation of representative sequencing libraries. Here we describe a protocol to capture nascent viral cDNA molecules, specifically the intermediates of the HIV-1 reverse transcription process.

The most critical step in this strategy is the ligation of an adaptor to the open 3'-termini in a quantitative and unbiased manner. Efficiencies of ligations between two ssDNA termini, both inter- and intramolecular, have been investigated and optimized for various applications^11^,^26^,^27^,^28^,^29^. The choice of using a hairpin adaptor with T4 DNA ligase under the conditions described in step 3.3 is the result of empirical optimization in which we evaluated different ligases, adaptors and reagents for the ligation of synthetic oligonucleotides representing HIV-1 sequences (Table 2) (data not shown). In these *in vitro* test reactions, we confirmed that the T4 DNA ligase mediated ligation of the hairpin adaptor, as described by Kwok *et al*.^11^, has a very low bias, and achieves near complete ligation of acceptor molecules when the adaptor is used in excess. The ligation efficiency was unaffected by the addition of nucleotide sequence to render the adaptor compatible for the multiplex primer system (see Figure 4). In comparison, we found that a thermostable 5'DNA/RNA ligase ("Ligase A", see Table of Materials for exact ligases compared here), which is an engineered RNA ligase that was developed in part to improve on ligation efficiency with ssDNA as the acceptor^27^, was indeed more effective at ligating two ssDNA molecules than RNA ligase ("Ligase B") but had a significant bias, with strong differences in ligation efficiency even between oligonucleotides with single base length differences [Table 2; HTP con mid G (a) and (b)]. Furthermore, we found only a minimal bias in reactions with "Ligase C" combined with an adaptor carrying a randomized 5'-termini (a strategy used to offset known nucleotide bias of "Ligase C"; see for example Ding *et al*.^30^). However, the "Ligase C"-mediated intermolecular ligations were incomplete, rendering the T4 DNA ligase system the superior choice.

Several quality control steps over the course of the protocol and the inclusion of positive and negative controls allow for the detection of potential problems before assay continuation and provide guidance for troubleshooting efforts. The qPCR quantifications in steps 2.2.2 and 2.3.12 ensure that the quantity of the input material is sufficient. Typical cDNA copy numbers in the 200 µL elution (from step 2.1) range from around 10,000 to 300,000 per µL. The hybrid capture step can result in some loss of overall HIV-1 cDNA quantity but should result in a strong enrichment of specific HIV-1 cDNA over cellular DNA, which can be determined by using appropriate primers to quantify genomic DNA before and after enrichment by qPCR or by measuring total DNA concentration. Recovered HIV-1 cDNA after the hybrid capture steps should be at least 10% of the input. Low starting material may otherwise explain a successful oligonucleotide positive control (see step 3.3.2) but only limited reads achieved in the samples. Low read numbers overall could also be explained by overestimation of the library concentration due to the presence of irrelevant DNA species without MiSeq adaptors. This would result in low cluster density and can be improved by determining the concentration of HIV-1 sequences in the library by qPCR in addition to the total DNA amount by fluorometric assays. Due to the highly sensitive nature of the method, special care should be taken to avoid even low-level contamination, both from other samples (in particular, from the high concentration control oligonucleotide stocks) as well as from laboratory equipment. Working in a UV sterilizing PCR workstation is beneficial in this regard. The automated gel electrophoresis of the final library (step 6.1.2) is a further quality control measure. The nucleic acid size range typically observed is between 150 to 500 nt. Primers that can be detected in the optional control after the PCR and before purification (see note in step 5.2) should now be absent. In a representative result, the sample intensity curve has a peak around 160 to 170 nt and a second sharper peak around 320 to 350 nt. This likely reflects the often-seen higher abundance in both relatively short (1 to 20 nt insert length) reverse transcripts and full-length strong-stop (180 to 182 nt insert length) (Figure 3b).

While the presented protocol and selected primers are specific for early HIV-1 reverse transcription constructs, the method is generally applicable to any study aiming to determine open 3'-termini of DNA. The main modifications required in other contexts will be the method for hybrid capture and the primer design strategy. For example, if the target is to be adapted to late HIV-1 transcripts, a larger number of different capturing biotinylated oligonucleotides annealing across the length of the cDNA would be advisable and will likely decrease the loss in the hybrid capture step. As mentioned in the introduction, it is important to consider limitations when designing the range over which 3'-termini are to be detected to avoid different sources of bias. First, there may be a bias in the PCR reactions if the templates with the adaptor are of vastly varying length. Second, the sequencing platform used here (*e.g.,* MiSeq) has a preferred insert length range for optimal clustering, and significantly shorter and longer products may not be sequenced with the same efficiency. In part, this can be addressed computationally, as was done by calculating a correction factor for linear length bias (see Figure 4, bottom graph). However, if the region of where 3'-termini mapping is desired is long (> 1000 nt), it is more advisable to split the reactions with the ligated transcripts and use multiple upstream primers to assess 3'-termini in sections.

The analysis program was written in-house for the specific purpose of analyzing both the last nucleotide of the HIV-1 sequence adjacent to the fixed adaptor sequence as well as the base variation of all bases to identify any mutations. The individual steps comprise the following: first, the adaptor sequences are trimmed using the fastx-0.0.13 toolkit; then, any sequences that are duplicated (meaning identical sequences including the barcode) are removed. All remaining unique reads are then aligned to the HIV-1 sequence using Bowtie (http://bowtie-bio.sourceforge.net/index.shtml) with the maximum mismatch set at three bases. The template sequence is comprised of the first 635 nt of HIV-1 cDNA (NL4.3 strain), which includes the -sss sequence and the first strand transfer product up to the polypurine track (U5-R-U3-PPT; see Figure 1). Thereby, the provided software and templates are only directly suitable if the method is used for the same application (detection of early reverse transcripts of the HIV-1_NL4.3_). Adjustments will have to be made for other target sequences. The positions of the 3'-termini for each read were determined by the position in the alignment. Base calls for each position are recorded and mutation rates are calculated from the total coverage of each base, which varies, as reads are of different lengths and long inserts may not be entirely covered by the 125-base sequencing in Read2.

To conclude, we believe the described method to be a valuable tool for many types of studies. Obvious applications include investigations of the mechanisms underlying reverse transcription inhibition through antiretroviral drugs or cellular restriction factors. However, only relatively minor adjustments should be necessary to adapt the system to 3'-termini mapping within other single-stranded DNA viral intermediates, which are present, for example, in parvovirus replication. Furthermore, the principle of the method, particularly its optimized ligation step, can provide a core part of library preparation design for the characterization of any 3'-DNA extensions, including elongations catalyzed by cellular double-stranded DNA polymerases.

## Supplementary Material

Table of Materials

## Figures and Tables

**Table 1:** Table of oligonucleotides including length, sequences, and modifications that are utilized in the described protocol. The table is adapted from a previous publication^6^. Please click here to download this table as an excel file.

Oligo name	Length in nt	Sequence	Purpose	Manufacturer (Purification)
full Kwok + MiSeq	61	5’-PHO-tgaagagcctagtcgctgttcannnnnnctgcccatagagagatcggaagagcacacgtct-SpC3-3’	Adaptor	IDT DNA technologies (HPLC)
2xBiotin SS bait	40	5’-biotin-cagtgtggaaaatctctagcagtggcgcccgaacagggac-biotin-3’	Hybrid Capture	MWG Eurofins (HPLC)
Biotin 1-16 ss	22	5’-cagtgtggaaaatctctagcag-BiTEG-3’	Hybrid Capture	MWG Eurofins (HPLC
Biotin tRNA + CTG	16	5’-cagtggcgcccgaaca-BITEG-3’	Hybrid Capture	MWG Eurofins (HPLC)
MP1.0 + 22HIV	82	5’-aatgatacggcgaccaccgagatctacactctttccctacacgacgctcttccgatctcactgctagagattttccacactg-3’	PCR amplification	MWG Eurofins (HPLC

**Table 2:** Table of 17 synthetic control oligonucleotides used as a positive control sample. The top 13 oligonucleotides were chosen based on size [long (116 to 120 nt), mid (69 to 85 nt), short (33 to 41 nt)] as well as their 3'-termini. The table is adapted from a previous publication^6^. Please click here to download this table as an excel file.

Oligo name	Length in nt	Sequence	Manufacturer (Purification)
HTP con long C	HTP con long C	120	5’-ctgctagagattttccacactgactaaaagggtctgagggatctctagttaccagagtcacacaacagacgggcacacactactttgagcactcaaggcaagctttattgaggcttaagc-3’	MWG Eurofins (HPLC)
HTP con long G	119	5’-ctgctagagattttccacactgactaaaagggtctgagggatctctagttaccagagtcacacaacagacgggcacacactactttgagcactcaaggcaagctttattgaggcttaag-3’	MWG Eurofins (HPLC)
HTP con long T	116	5’-ctgctagagattttccacactgactaaaagggtctgagggatctctagttaccagagtcacacaacagacgggcacacactactttgagcactcaaggcaagctttattgaggctt-3’	MWG Eurofins (HPLC)
HTP con long A	118	5’-ctgctagagattttccacactgactaaaagggtctgagggatctctagttaccagagtcacacaacagacgggcacacactactttgagcactcaaggcaagctttattgaggcttaa-3’	MWG Eurofins (HPLC)
HTP con mid C	76	5’-ctgctagagattttccacactgactaaaagggtctgagggatctctagttaccagagtcacacaacagacgggcac-3’	MWG Eurofins (HPLC)
HTP con mid G (a)	71	5’-ctgctagagattttccacactgactaaaagggtctgagggatctctagttaccagagtcacacaacagacg-3’	MWG Eurofins (HPLC)
HTP con mid G (b)	72	5’-ctgctagagattttccacactgactaaaagggtctgagggatctctagttaccagagtcacacaacagacgg-3’	MWG Eurofins (HPLC)
HTP con mid A	69	5’-ctgctagagattttccacactgactaaaagggtctgagggatctctagttaccagagtcacacaacaga-3’	MWG Eurofins (HPLC)
HTP con mid T	85	5’-ctgctagagattttccacactgactaaaagggtctgagggatctctagttaccagagtcacacaacagacgggcacacactactt-3’	MWG Eurofins (HPLC)
HTP con short A	40	5’-ctgctagagattttccacactgactaaaagggtctgaggga-3’	MWG Eurofins (HPLC)
HTP con short T	33	5’-ctgctagagattttccacactgactaaaagggt-3’	MWG Eurofins (HPLC)
HTP con short G	41	5’-ctgctagagattttccacactgactaaaagggtctgaggg-3’	MWG Eurofins (HPLC)
HTP con short C	34	5’-ctgctagagattttccacactgactaaaagggtc-3’	MWG Eurofins (HPLC)
HTP Con 46 (T)	46	5’-ctgctagagattttccacactg actaaaagggtctgagggatctct-3’	MWG Eurofins (HPLC)
HTP Con 83 (C)	83	5’-ctgctagagattttccacactg actaaaagggtctgagggatctctagttaccagagtcacacaacagacgggcacacactac-3’	MWG Eurofins (HPLC)
HTP Con 103 (C)	103	5’-ctgctagagattttccacactg actaaaagggtctgagggatctctagttaccagagtcacacaacagacgggcacacactactttgagcactcaaggcaagc-3’	MWG Eurofins (HPLC)
HTP Con 107 (A)	107	5’-ctgctagagattttccacactg actaaaagggtctgagggatctctagttaccagagtcacacaacagacgggcacacactactttgagcactcaaggcaagcttta-3’	MWG Eurofins (HPLC)
