# Supplementary material for: Determining 3'-Termini and Sequences of Nascent Single-Stranded Viral DNA Molecules during HIV-1 Reverse Transcription in Infected Cells
Source: J Vis Exp. Author manuscript; Available in PMC 2019 Aug 5. (PMC6682491; doi:10.3791/58715)
Supplement: Table of Materials [file EMS82915-supplement-Table_of_Materials.pdf]

Materials List for:

# Determining 3'-Termini and Sequences of Nascent Single-Stranded Viral DNA Molecules during HIV-1 Reverse Transcription in Infected Cells

Darja Pollpeter[1], Andrew Sobala[1], Michael H. Malim[1]

[1]Department of Infectious Diseases, School of Immunology & Microbial Sciences, King's College London

Correspondence to: Darja Pollpeter at darja.pollpeter@kcl.ac.uk

URL: https://www.jove.com/video/58715
DOI: doi:10.3791/58715

## Materials

| Name | Company | Catalog Number | Comments |
|---|---|---|---|
| 293T cells | ATCC | CRL-3216 | |
| Dulbecco's Modified Eagle's Medium | Gibco | 31966-021 | |
| Penicillin/Streptomycin | Gibco | 15150-122 | |
| Fetal Bovine Serum | Gibco | 10270-106 | |
| HeraCell Vios 250i CO2 Incubator | Thermo Scientific | 51030966 | |
| Laminar flow hood - CAS BioMAT2 | Wolflabs | CAS001-C2R-1800 | |
| 10mm TC-treated culture dish | Corning | 430167 | |
| TrypLE™ Express (1x), Stable Trypsin Replacement Enzyme | Gibco | 12605-010 | |
| OptiMEM® (Minimal Essential Medium) | Gibco | 31985-047 | |
| HIV-1 NL4-3 Infectious Molecular Clone (pNL4-3) | NIH Aids reagent program | 114 | |
| Polyethylenimine (PEI) - MW:25000 | PolySciences Inc | 23966-2 | dissolved at 1mg/ml and adjusted to pH7 |
| RQ1- Rnase free Dnase | Promega | M6101 | |
| Filter 0.22 µm | Triple Red Limited | FPE404025 | |
| 15 mL polypropylene tubes | Corning | CLS430791 | |
| Sucrose | Calbiochem | 573113 | |
| Phosphate Buffered Saline (1x) | Gibco | 14190-094 | |
| Ultracentrifuge tubes | Beckman Coulter | 344060 | |
| Ultracentrifuge | Sorval | WX Ultra Series | Th-641 Rotor |
| Alliance HIV-1 p24 antigen ELISA kit | Perkin Elmer | NEK050001KT | |
| CEM-SS cells | NIH Aids reagent program | 776 | |
| Roswell Park Memorial Institute Medium | Gibco | 31870-025 | |
| CoStar® TC treated multiple well plates | Corning | CLS3513-50EA | |
| Benchtop centrifuge: Heraus™ Multifuge™ X3 FR | Thermo Scientific | 75004536 | |
| TX-1000 Swinging Bucket Rotor | Thermo Scientific | 75003017 | |
| Microcentrifuge: 5424R | Eppendorf | 5404000060 | |
| Total DNA extraction kit (DNeasy Blood and Tissue kit) | Qiagen | 69504 | |
| Nuclease free H2O | Ambion | AM9937 | |

Copyright © 2019 Creative Commons Attribution 3.0 License

| | | | |
|---|---|---|---|
| Cutsmart buffer | New England Biolabs (part of DpnI enzyme) | R0176S | |
| DpnI restriction enzyme | New England Biolabs | R0176S | |
| Oligonucleotides for qPCR | MWG Eurofins | N/A | HPSF purification |
| TaqMan PCR Universal Mastermix | Thermo | 4304437 | |
| LoBind Eppendorf® tubes | Eppendorf | 30108078 | |
| Axygen™ aerosol filter pipette tips, 1000 µL | Fisher Scientific | TF-000-R-S | |
| Axygen™ aerosol filter pipette tips, 200 µL | Fisher Scientific | TF-200-R-S | |
| Axygen™ aerosol filter pipette tips, 20 µL | Fisher Scientific | TF-20-R-S | |
| Axygen™ aerosol filter pipette tips, 10 µL | Fisher Scientific | TF-10-R-S | |
| PCR clean hood | LabCaire | Model PCR-62 | |
| DynaMag™2-magnet | Thermo | 12321D | |
| Streptavidin MagneSphere® paramagnetic particles | Promega | Z5481 | |
| Casein | Thermo Scientific | 37582 | |
| End over end rotator, Revolver™ 360° | Labnet | H5600 | |
| Tris-Base | Fisher Scientific | BP152-5 | |
| Hydrochloric Acid | Sigma | H1758-100ML | |
| EDTA disodium salt dihydrate | Electran (VWR) | 443885J | |
| Sodium Chloride | Sigma | S3014 | |
| Dri-Block® Analog Block Heater | Techne | UY-36620-13 | |
| PCR tubes and domed caps | Thermo Scientific | AB0266 | |
| PCR machine | Eppendorf | Mastercycler® series | |
| T4 DNA ligase | New England Biolabs | M0202M | |
| 40% Polyethylene glycol solution (PEG) in H2O, MW: 8000 | Sigma | P1458-25ML | |
| Betaine solution, 5M | Sigma | B0300-1VL | |
| Gel loading buffer II (formamide buffer) | Thermo Scientific | AM8546G | |
| Precast 6% TBE urea gels | Invitrogen | EC6865BOX | |
| Mini cell electrophoresis system | Invitrogen, Novex | XCell SureLock™ | |
| Tris/Borate/EDTA solution (10x) | Fisher Scientific | 10031223 | |
| Needle 21 G x1 1/2 | VWR | 613-2022 | |
| SYBR Gold nucleic acid stain (10000x) | Life Technologies | S11494 | |
| Dark Reader DR46B transilluminator | Fisher Scientific | NC9800797 | |
| Ammonium acetate | Merck | 101116 | |
| SDS solution 20% (w/v) | Biorad | 161-0418 | |
| Centrifuge tube filter | Appleton Woods | BC591 | |
| Filter Glass Fibre Gf/D 10mm | Whatman (VWR) | 512-0427 | |
| polyadenylic acid (polyA) RNA | Sigma | 10108626001 | |
| Glycogen, molecular biology grade | Thermo Scientific | R0561 | |
| Isopropanol (2-propanol) | Fisher Scientific | 15809665 | |

Copyright © 2019 Creative Commons Attribution 3.0 License

| | | | |
|---|---|---|---|
| Ethanol, molecular biology grade | Fisher Scientific | 10041814 | |
| Accuprime™ Supermix I (DNA polymerase premix) | Life Technologies | 12342-010 | |
| NEBNext® Multiplex Oligo for Illumina (Index Primer Set 1 and 2) | New England Biolabs | E7335S; E7500S | |
| Tapestation D1000 Screentape High sensitivity | Agilent Technologies | 5067- 5584 | |
| Tapestation D1000 Reagents | Agilent Technologies | 5067- 5585 | |
| 2200 Tapestation - automated gel electrophoresis system | Agilent Technologies | G2965AA | |
| Agencourt® AMPure® beads XP | Beckman Coulter | A63880 | |
| Qubit™ dsDNA HS Assay Kit | Invitrogen | Q32851 | |
| Qubit™ 2.0 Fluorometer | Invitrogen | Q32866 | |
| Topo™ TA cloning Kit | Invitrogen | 450071 | |
| Sequencing platform: MiSeq System | Illumina | | |
| Experiment Manager (Sample sheet software) | Illumina | Note: Use TruSeq LT as a template | |
| Miseq™ Reagent kit V3 (150 cycle) | Illumina | MS-102-3001 | |
| Sequencing hub: Basespace | Illumina | https://basespace.illumina.com | |
| *Ligase A: Thermostable 5' App DNA/RNA ligase* | *NEB* | *M0319S* | Not used in this protocol, but tested in optimization process with results described in the discussion. |
| *Ligase B: T4 RNA ligase 1* | *NEB* | *M0204* | Not used in this protocol, but tested in optimization process with results described in the discussion. |
| *Ligase C: CircLigase* | *Epicentre* | *CL4111K* | Not used in this protocol, but tested in optimization process with results described in the discussion. |

Copyright © 2019 Creative Commons Attribution 3.0 License
